# Transcriptomic and proteomic analysis of tumor suppressive effects of GZ17-6.02 against mycosis fungoides

**DOI:** 10.1038/s41598-024-52544-z

**Published:** 2024-01-23

**Authors:** Zachary A. Bordeaux, Sriya V. Reddy, Justin Choi, Gabriella Braun, Jaimie McKeel, Weiying Lu, Selina M. Yossef, Emily Z. Ma, Cameron E. West, Shawn G. Kwatra, Madan M. Kwatra

**Affiliations:** 1 Department of Dermatology, Johns Hopkins University School of Medicine, Cancer Research Building II, Suite 206 1550 Orleans Street, Baltimore, MD 21231 USA; 2 Department of Anesthesiology, Duke University School of Medicine, Durham, USA; 3 Genzada Pharmaceuticals, Hutchinson, USA; 4 US Dermatology Partners, Wichita, USA; 5 Department of Oncology, Johns Hopkins University School of Medicine, Baltimore, USA; 6 Department of Pharmacology and Cancer Biology, Duke University School of Medicine, Durham, USA

**Keywords:** Cancer therapy, Skin cancer, Cancer, Proteomics, Sequencing

## Abstract

Mycosis fungoides (MF) is the most common form of cutaneous T-cell lymphoma (CTCL). Despite having a wide variety of therapeutic agents available for the treatment of MF, patients often suffer from a significant decrease in quality of life and rarely achieve long-term remission or complete cure, highlighting a need to develop novel therapeutic agents for this disease. The present study was undertaken to evaluate the efficacy of a novel anti-tumor agent, GZ17-6.02, which is composed of curcumin, harmine, and isovanillin, against MF in vitro and in murine models. Treatment of HH and MyLa cells with GZ17-6.02 inhibited the growth of both cell lines with IC50 ± standard errors for growth inhibition of 14.37 ± 1.19 µg/mL and 14.56 ± 1.35 µg/mL, respectively, and increased the percentage of cells in late apoptosis (*p* = .0304 for HH; *p* = .0301 for MyLa). Transcriptomic and proteomic analyses revealed that GZ17-6.02 suppressed several pathways, including tumor necrosis factor (TNF)-ɑ signaling via nuclear factor (NF)-kB, mammalian target of rapamycin complex (mTORC)1, and Pi3K/Akt/mTOR signaling. In a subcutaneous tumor model, GZ17-6.02 decreased tumor volume (*p* = .002) and weight (*p* = .009) compared to control conditions. Proteomic analysis of tumor samples showed that GZ17-6.02 suppressed the expression of several proteins that may promote CTCL growth, including mitogen-activated protein kinase (MAPK)1, MAPK3, Growth factor receptor bound protein (GRB)2, and Mediator of RAP80 interactions and targeting subunit of 40 kDa (MERIT)40.

## Introduction

Mycosis fungoides (MF) is the most common form of cutaneous T-cell lymphoma (CTCL) and is characterized by clonal proliferations of skin-homing helper T cells with a predilection for epidermal involvement^1,2^. It typically presents as persistent, progressive, erythematous patches and plaques but can progress to advanced stages where patients may develop tumors, ulceration, and systemic involvement^3–5^. Although MF typically follows an indolent course with a median survival rate of 20–35 years in its early stages^3,6^, patients often experience a significant decrease in quality of life secondary to psychosocial dysfunction, a high cost of healthcare utilization, intense pruritus, and sleep disturbances^7–12^. In the early stages, treatment of MF revolves around controlling cutaneous lesions using modalities such as topical corticosteroids, topical nitrogen mustards, topical retinoids, phototherapy, and radiation therapy^1,2,13,14^. As the disease progresses or becomes refractory to topical agents, systemic therapies are utilized. They include oral retinoids, interferons, histone deacetylase inhibitors, monoclonal antibodies against cluster of differentiation (CD)52 and C–C chemokine receptor (CCR)4, extracorporeal photopheresis, and chemotherapeutic agents^1,2,13,14^. Despite the wide variety of therapeutic agents available for the treatment of MF, patients rarely achieve long-term remission or complete cure, highlighting a need to develop novel therapeutic agents for this disease^15–18^.

The present study was undertaken to evaluate GZ17-6.02, a novel anti-cancer agent currently undergoing clinical trials for various solid malignancies (NCT03775525), against in vitro and in vivo models of MF. The rationale for evaluating GZ17-6.02, which is a combination agent composed of curcumin (10% by weight), harmine (13% by weight), and isovanillin (77% by weight)^19^, for the treatment of MF, was based on its activity against a variety of neoplasms both in vitro and in murine models, including head and neck squamous cell carcinoma, breast cancer, renal carcinoma, pancreatic ductal adenocarcinoma, melanoma, glioblastoma, and actinic keratoses^19–26^.

The anti-tumor properties of GZ17-6.02 appear to be due to an inherent synergism of its components which have been shown to affect a wide variety of targets. Curcumin is a component of the dietary spice turmeric that has demonstrated anti-neoplastic effects through inhibition of the phosphoinositide 3-kinase (Pi3K)-Akt pathway, epidermal growth factor receptor (EGFR) signaling, vascular endothelial growth factor (VEGF), and matrix metalloproteases, as well as by promoting caspase and mitochondria-driven apoptosis^27–32^. Additionally, curcumin has been shown to induce apoptosis in CTCL cells, believed to be due to the inhibition of nuclear factor (NF)-κB and signal transducer and activator of transcription (STAT)3 signaling^33,34^. Similarly, harmine is shown to inhibit extracellular signal-regulated kinase (ERK) and Akt signaling and to disrupt EGFR activity by suppressing the dual-specificity tyrosine-(Y)-phosphorylation-regulated kinase, DYRK1A^35–37^. Harmine also exerts anti-tumor activity by blocking the epithelial-to-mesenchymal transition, suppressing angiogenesis, inducing DNA damage, and inhibiting DNA replication^38–40^.

The purpose of this study was to evaluate the ability of GZ17-6.02 to inhibit the growth of MF cell lines in vitro and in a murine model, as well as to further clarify its mechanism of action using transcriptomic and proteomic approaches. Given its reported efficacy against a wide variety of neoplastic conditions^19–26^, we hypothesize that the medication will exert cytotoxic effects against MF cells through the induction of apoptosis and inhibition of targets related to the Pi3K-Akt and EGFR signaling pathways. Our results show that GZ17-6.02 inhibits the growth of MF cells in vitro while also exerting anti-tumor activity in vivo. The underlying mechanism, revealed by transcriptomic and proteomic analyses, involves its action at multiple levels, including the downregulation of pathways such as Pi3K-Akt, MAPK, and NF-κB signaling.

## Materials and methods

### Ethical statement

All animal protocols used in this study were approved and performed in accordance with guidelines set forth by the Duke University Institutional Animal Care & Use Committee (IACUC) under protocol number A155-20-07. All experiments were performed following the ARRIVE guidelines (http://arriveguidelines.org) to report animal experiments.

### Compounds and reagents

For in vitro experiments, GZ17-6.02 was dissolved in dimethyl sulfoxide (DMSO) as a stock solution and stored at − 20 °C. Working solutions were diluted in fresh medium not exceeding a final DMSO concentration of 1%. All compounds were provided by Genzada Pharmaceuticals (Sterling, KS).

### Cell culture

HH (ATCC, #CRL-2105) and MyLa 2059 (MyLa) (University of Copenhagen, Denmark) cells were cultured in RPMI-1640 (Gibco #11875093) supplemented with 10% fetal bovine serum. Cells were maintained at a concentration of 1 × 10^5^ to 1 × 10^6^ cells per ml. For experiments, only cells with less than five passages were used.

### Cell viability assays

Various concentrations of compounds were plated onto 384-well plates (Corning, #3764) using an Echo® 550 Liquid Handler (Labcyte, #001-16079). A Matrix Wellmate Microplate Dispenser (Thermo Scientific, #201-30002) was used to dispense HH and MyLa cells (1000 cells per well) onto the pre-plated compounds for a final reaction volume of 25 µL and final DMSO concentration of 1%. The plates were incubated (37ºC, 5% CO_2_, 95% humidity) for 72 h and assayed using CellTiter Glo® reagent (Promega, #G7570). Luminescence values were recorded using a Clariostar Microplate reader (BMG Labtech, #0430B0001B), and the data were analyzed using GraphPad Prism 9.0 Software (San Diego, CA).

### Flow cytometry analysis of apoptosis

HH and MyLa cells were treated with GZ17-6.02 (25 µg/mL or 50 µg/mL in 0.5% DMSO) for 1 h. Apoptosis was evaluated using an Annexin-V/propidium iodide double staining assay kit (Sigma Aldrich, #APOAF) according to the manufacturer’s instructions. Cellular fluorescence was measured using a BD FACS Canto flow cytometer (BD Biosciences, #BF-FACSC2). Cells in early apoptosis were Annexin-V positive and propidium iodide negative, while cells in late apoptosis were both Annexin-V and propidium iodide positive.

### Western blot

HH and MyLa cells were treated with vehicle or GZ17-6.02 (12.5 or 25 μg/ml), collected, washed with ice-cold phosphate-buffered saline (PBS), lysed with lysis buffer (1% Triton X‐100, 50 mM HEPES, pH 7.4, 150 mM NaCl, 1.5 mM MgCl_2_, 1 mM EGTA, 100 mM NaF, 10 mM Na pyrophosphate, 1 mM Na_3_VO_4_, 10% glycerol, protease inhibitor (Roche Applied Science, #05056489001) and phosphatase inhibitor (Roche Applied Science, #04906837001)) for 30 min, and clarified by centrifugation at 10,000 r.p.m. A Bradford colorimetric protein assay (Bio-Rad, Hercules, CA) was used to quantify protein. Protein extracts were loaded on a 4–12% SDS-PAGE Bis–Tris gel (Thermofisher, #NP0322) and transferred onto polyvinylidene difluoride (PVDF) membranes (Invitrogen, Waltham, MA). Membranes were blocked with 5% milk in tris-buffered saline (TBS) and 0.1% Tween-20 (TBS-T) at room temperature for one hour and washed with TBS-T three times. The membranes were then incubated with primary antibodies against B-actin (Cell Signaling, #4970) and cleaved poly (ADP-ribose) polymerase (PARP) (Cell Signaling, #5625) at a ratio of 1:1000 in TBS-T with 5% bovine serum albumin (BSA) overnight at 4 °C. After the incubation, membranes were washed with TBS-T three times and incubated with horseradish peroxidase (HRP)-labeled secondary antibodies (Santa Cruz, #SC-2357) at a ratio of 1:1000 in 5% milk in TBS-T for one hour at room temperature. The membrane was developed on Biomax MR film. Unedited western blot images are provided in Supplemental Figs. S1, S2. 

### mRNA sequencing

HH and MyLa cells were treated with GZ17-6.02 (25 µg/mL in 0.5% DMSO) or vehicle for 24 h. Total RNA was isolated using an RNAeasy plus kit (Qiagen, #74034) according to the manufacturer’s instructions. The TrimGalore toolkit (https://www.bioinformatics.babraham.ac.uk/projects/trim_galore) was used to process RNA sequencing (RNAseq) data. Only reads that were 20nt or longer after trimming were used for further analysis. Reads were mapped to the GRCh38v93 version of the human genome and transcriptome^41^ using the STAR RNA-seq alignment tool^42^. Reads were kept for subsequent analysis if they mapped to a single genomic location. Gene counts were compiled using the HTSeq tool (http://www.huber.embl.de/users/anders/HTSeq). Only genes with at least 10 reads in any given library were used in subsequent analyses. Normalization and differential expression were carried out using the DESeq2^43^ Bioconductor^44^ package with the R statistical programming environment (www.r-project.org). The false discovery rate was calculated to control for multiple hypothesis testing. Gene set enrichment analysis (GSEA) was performed using the GSEA software^45,46^ with the Hallmark database as reference^47^. Gene set variation analysis (GSVA) was conducted with the GSVA R Bioconductor package using the R statistical programming environment^48^. Differentially expressed genes (DEGs) were defined as coding genes with a log fold change > 1 or < − 1 and a false discovery rate-adjusted *p* value < 0.05.

### Reverse phase protein array (RPPA)

For in vitro experiments, HH and MyLa cells were treated with GZ17-6.02 (25 µ*g*/mL in 0.5% DMSO) or vehicle for 24 h. Cell lysates were prepared as described above for the western blotting. After quantifying protein concentration, lysates were denatured with 4× sodium dodecyl sulfate (SDS) sample buffer (40% glycerol, 8% SDS, 0.25 M Tris–HCl, pH 6.8, 10% (v/v) 2-mercaptoethanol) and boiled for 5 min. Samples were stored at − 80 °C and then sent to MD Anderson’s RPPA core facility (Houston, TX). For in vivo experiments, tumor samples were collected from mice, flash-frozen in liquid nitrogen, and stored at − 80 °C prior to being sent to MD Anderson’s RPPA core facility for processing. Hallmark functional enrichment analyses were performed on gene lists of proteins or phosphoproteins whose expression decreased with GZ17-6.02 treatment using EnrichR^49–51^.

### Animal experiments

A subcutaneous xenograft model was used to study the effects of GZ17-6.02 in vivo^52–54^. Male NOD.SCID.gamma (NSG) mice aged 8–12 weeks weighing 20–25 g were obtained from the breeding core at Duke University Medical Center. Mice were housed in IACUC-compliant Allentown 75 JAG cages. Up to 4 mice were housed in a single cage and were provided with constant access to food and water. Mice were inoculated with 1 × 10^6^ freshly dissociated HH cells suspended in 200 µL media subcutaneously into the right flank. Then, 72 h after tumor inoculation, mice were randomized into vehicle (*n* = 8) and treatment (*n* = 8) groups. Vehicle control animals received 200 µL Peptamen daily by oral gavage. Mice in the treatment arm received 300 mg/kg GZ17-6.02 daily by oral gavage. At this dose of GZ17-6.02, no adverse effects of the drug were seen. Animals were observed daily, body mass was measured once weekly, and tumor volumes were measured every two days with hand-held Vernier calipers. Tumor volume was calculated with the formula V = (width^2^) × (length)/2. Animals were euthanized using carbon dioxide to minimize suffering after 30 days of treatment.

#### Statistical analysis

Statistical analyses were conducted using GraphPad Prism 9.0. *p* values for GSEA were adjusted using the Benjamini–Hochberg method. Comparisons between all other groups were made using the Student’s *t*-test. Differences with a *p* value or adjusted *p* value of < 0.05 were considered statistically significant.

## Results

### GZ17-6.02 suppresses the viability of mycosis fungoides cells

**Figure 1:**
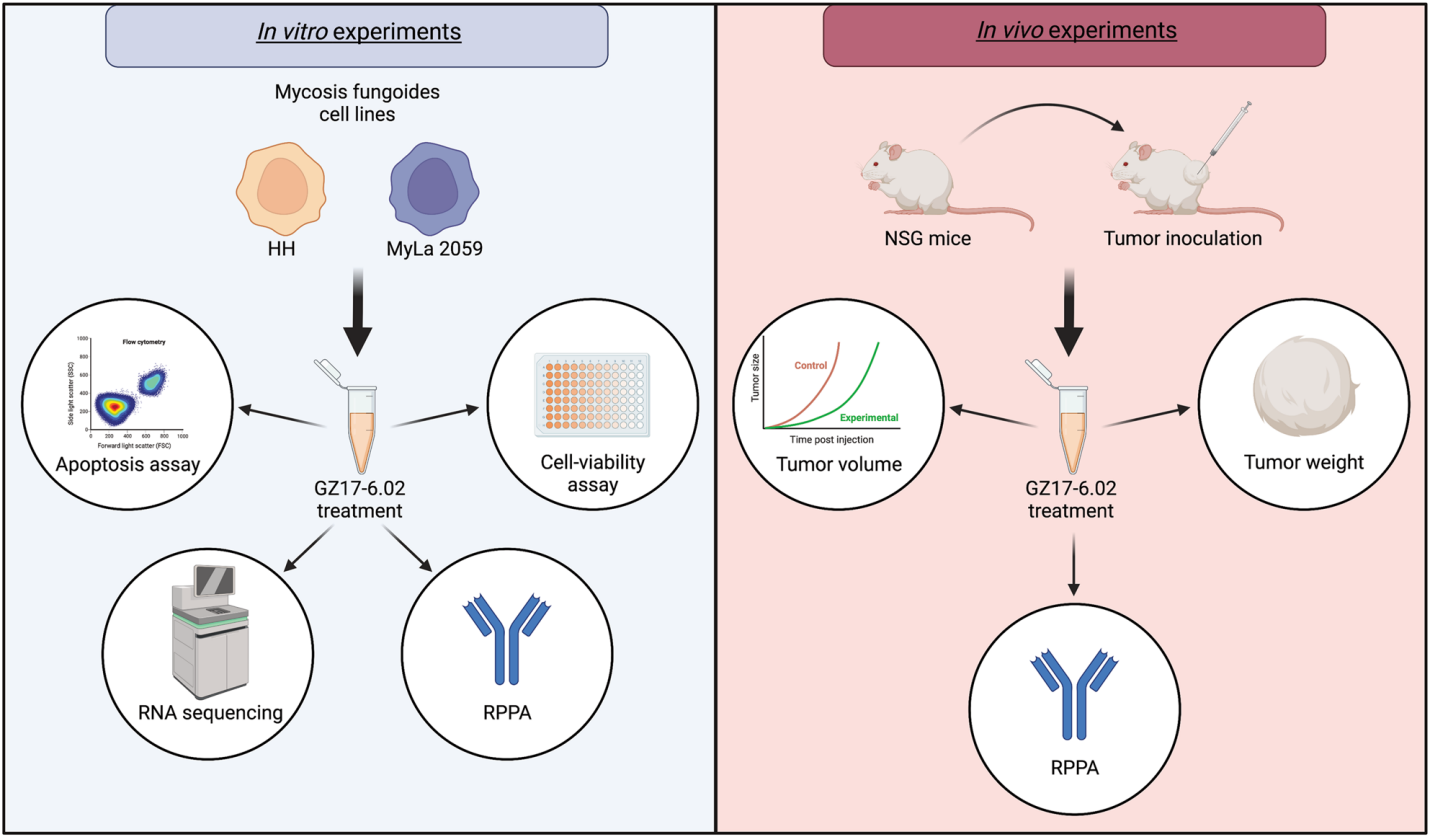
Overall study design. HH and MyLa 2059 cells were used for in vitro experiments. Treated cells were subjected to cell viability assays, apoptosis assays, RNA sequencing, and RPPA. NSG mice were used for in vivo models of MF. Mice were inoculated with HH cells subcutaneously and treated with GZ17-6.02 or vehicle. Tumor volume was measured over time, and at the end of the experiment, tumors were extracted, weighed, and prepared for RPPA. RPPA, reverse phase protein arrays; NSG, NOD.SCID.gamma.

**Figure 2:**
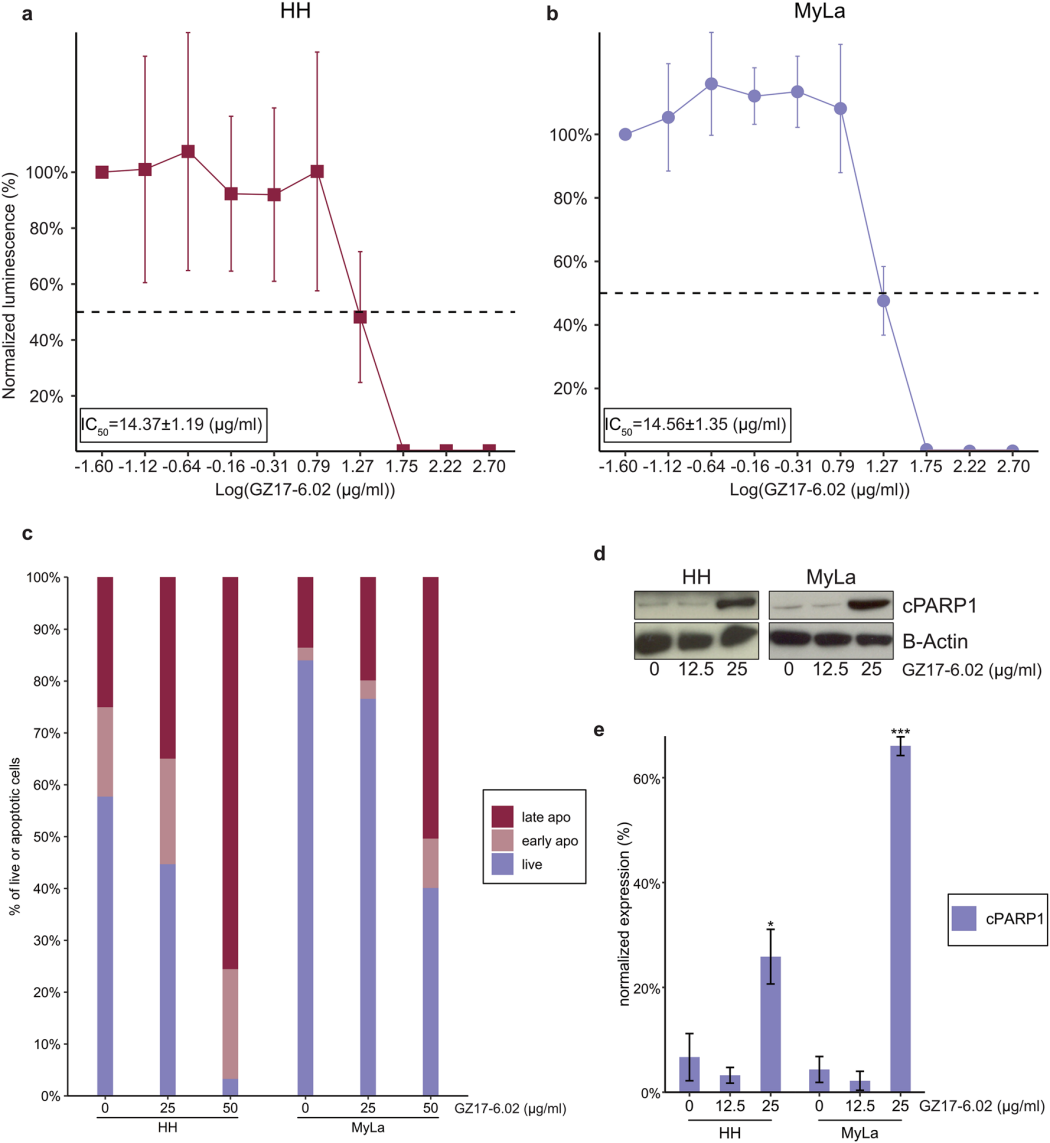
GZ17-6.02 inhibits the growth of MF cells. (**A**) Representative dose–response curve for GZ17-6.02 against HH cells. (**B**) Representative dose–response curve for GZ17-6.02 against MyLa cells. (**C**) Percentage of HH and MyLa cells in early and late apoptosis following incubation with increasing concentrations of GZ17-6.02. (**D**) Western blot of cleaved PARP in HH and MyLa cells following incubation with increasing concentrations of GZ17-6.02. (**E**) Normalized expression of western blot experiments showing increased cleaved PARP with increasing concentrations of GZ17-6.02 in HH and MyLa cells. The blots for HH and MyLa show in this figure were taken from separate gels. *p < .05; **p < .01; ***p < .001; apo, apoptosis; cPARP, cleaved PARP.

The overall study design is shown in Fig. 1. In order to assess the potential of GZ17-6.02 as a therapeutic agent for MF, we first performed a cell viability assay on treated HH and MyLa cells to determine its ability to suppress MF growth and produce cell death in vitro. As can be seen in Fig. 2A,B, GZ17-6.02 inhibited the growth of both cell lines with IC_50_ ± standard errors for growth inhibition of 14.37 ± 1.19 µg/mL for HH and 14.56 ± 1.35 µg/mL for MyLa.

Given the observed cytotoxic effects of GZ17-6.02 against MF, we next sought to determine if the cell death observed in the viability assay was mediated by the induction of apoptosis using flow cytometric analysis of apoptosis on control and treated HH and MyLa cells (Fig. 2C). The mean ± standard deviation percentage of HH cells in late apoptosis increased from 17.33 ± 5.23% with control conditions to 65.57 ± 12.25% with the addition of 50 µg/mL GZ17-6.02 (*p* = 0.0304). For MyLa cells, the addition of 50 µg/mL GZ17-6.02 resulted in an increase in the percentage of cells in early apoptosis from 2.20 ± 1.20% with control conditions to 8.40 ± 1.79% (*p* = 0.0153) and an increase in the percentage of cells in late apoptosis from 12.17 ± 3.92% with control conditions to 44.40 ± 6.65% (*p* = 0.0301). To confirm the pro-apoptotic effects of GZ117-6.02 we next utilized western blots to evaluate cleaved PARP (cPARP) levels with varying concentrations of GZ17-6.02 (Fig. 2D,E). PARP was chosen for these experiments as its cleavage by caspases is considered to be one of the hallmarks of apoptosis^55,56^. With the addition of 25 µ*g*/mL GZ17-6.02, the mean ± standard deviation normalized expression of cPARP increased from 6.69 ± 4.50% with control conditions to 25.86 ± 5.21% (*p* = 0.017) for HH and 4.34 ± 2.47% with control conditions to 66.07 ± 1.85% for MyLa (*p* < 0.001). These findings suggest that cleavage of PARP may mediate the pro-apoptotic effects of GZ17-6.02.

### Transcriptomic and proteomic effects of GZ17-6.02 in vitro

**Figure 3:**
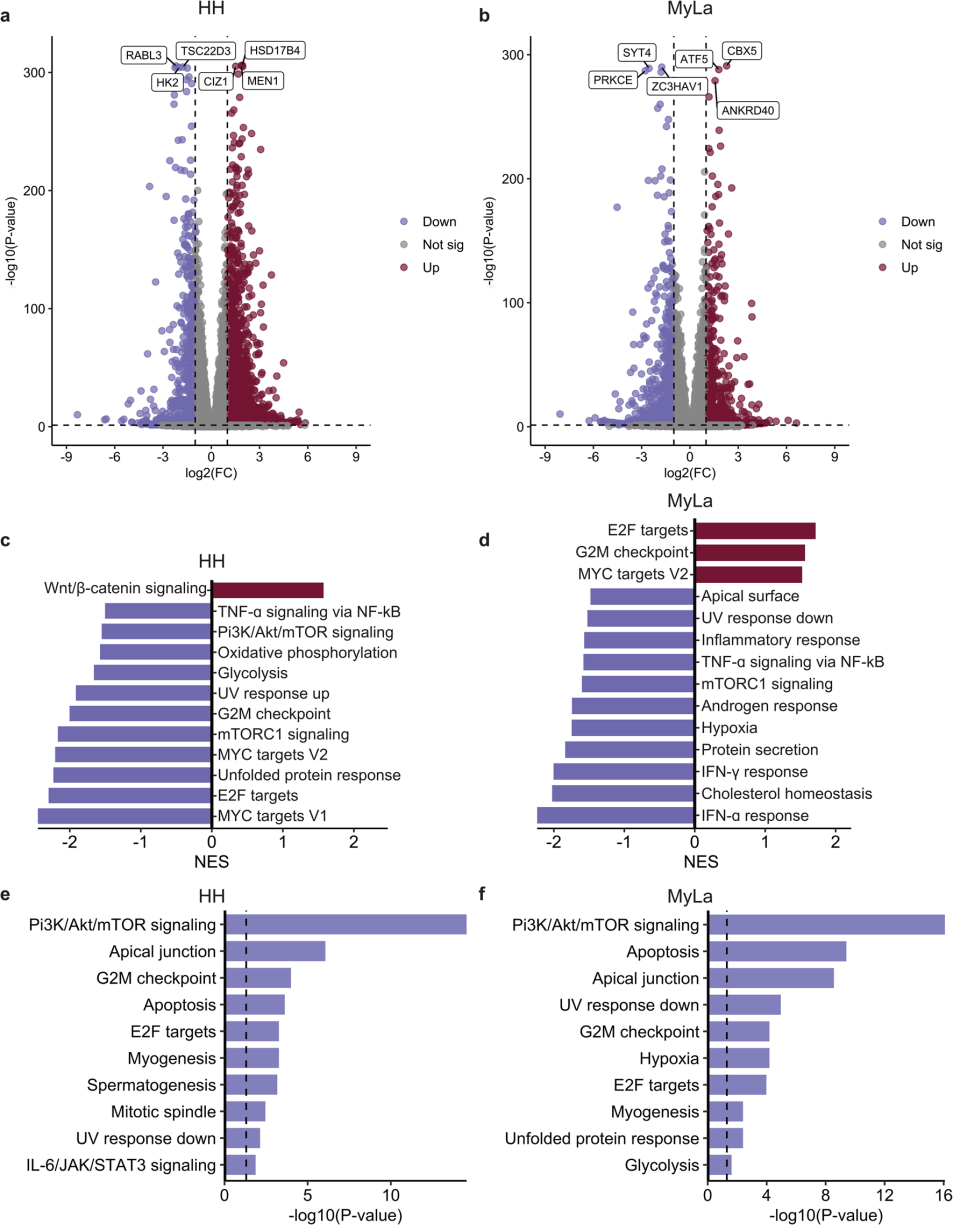
Molecular mechanism of GZ17-6.02 in vitro. (**A**) Volcano plot of DEGs between control and GZ17-6.02 treated HH cells. (**B**) Volcano plot of DEGs between control and GZ17-6.02 treated MyLa cells. (**C**) GSEA results showing Hallmark pathways upregulated and downregulated in HH cells following GZ17-6.02 treatment. (**D**) GSEA results showing Hallmark pathways upregulated and downregulated in MyLa cells following GZ17-6.02 treatment. (**E**) Top downregulated pathways in GZ17-6.02 treated HH cells from Hallmark EnrichR enrichment analysis of phosphor-proteomic data. (**F**) Top downregulated pathways in GZ17-6.02 treated MyLa cells from Hallmark EnrichR enrichment analysis of phosphor-proteomic data. FC, fold change; sig, significant; TNF, tumor necrosis factor; UV, ultraviolet; IFN, interferon; NES, normalized enrichment score.

Given the observed cytotoxic effects of GZ17-6.02 against MF cells, we next performed RNAseq on control and treated HH and MyLa cells to gain a better understanding of its mechanism of action. Differential expression analysis of transcriptomic data revealed 2951 DEGs (2191 upregulated and 780 downregulated) between control and GZ17-6.02 treated HH cells (Fig. 3A), and 1882 DEGs, (923 upregulated and 959 downregulated) between control and treated MyLa cells (Fig. 3B). Pathway analysis with GSEA was performed on differential expression analysis data to gain a broader understanding of the pathways effected by GZ17-6.02. This analysis revealed downregulation of many pathways in GZ17-6.02 treated cells, including tumor necrosis factor (TNF)-ɑ signaling via NF-kB and mammalian target of rapamycin complex (mTORC)1 signaling in both cell lines and Pi3K/Akt/mammalian target of rapamycin (mTOR) signaling in HH alone (Fig. 3C,D). We next conducted RPPA on control and GZ17-6.02 treated cells further clarify the compounds mechanism of action and see if similar targets identified in our transcriptomic analyses were affected at a proteomic level. Pathway analysis using EnrichR on gene lists of phosphoproteins downregulated by GZ17-6.02 also revealed suppression of Pi3K/Akt/mTOR signaling in both cell lines in addition to several other pathways (Fig. 3E,F).

**Figure 4:**
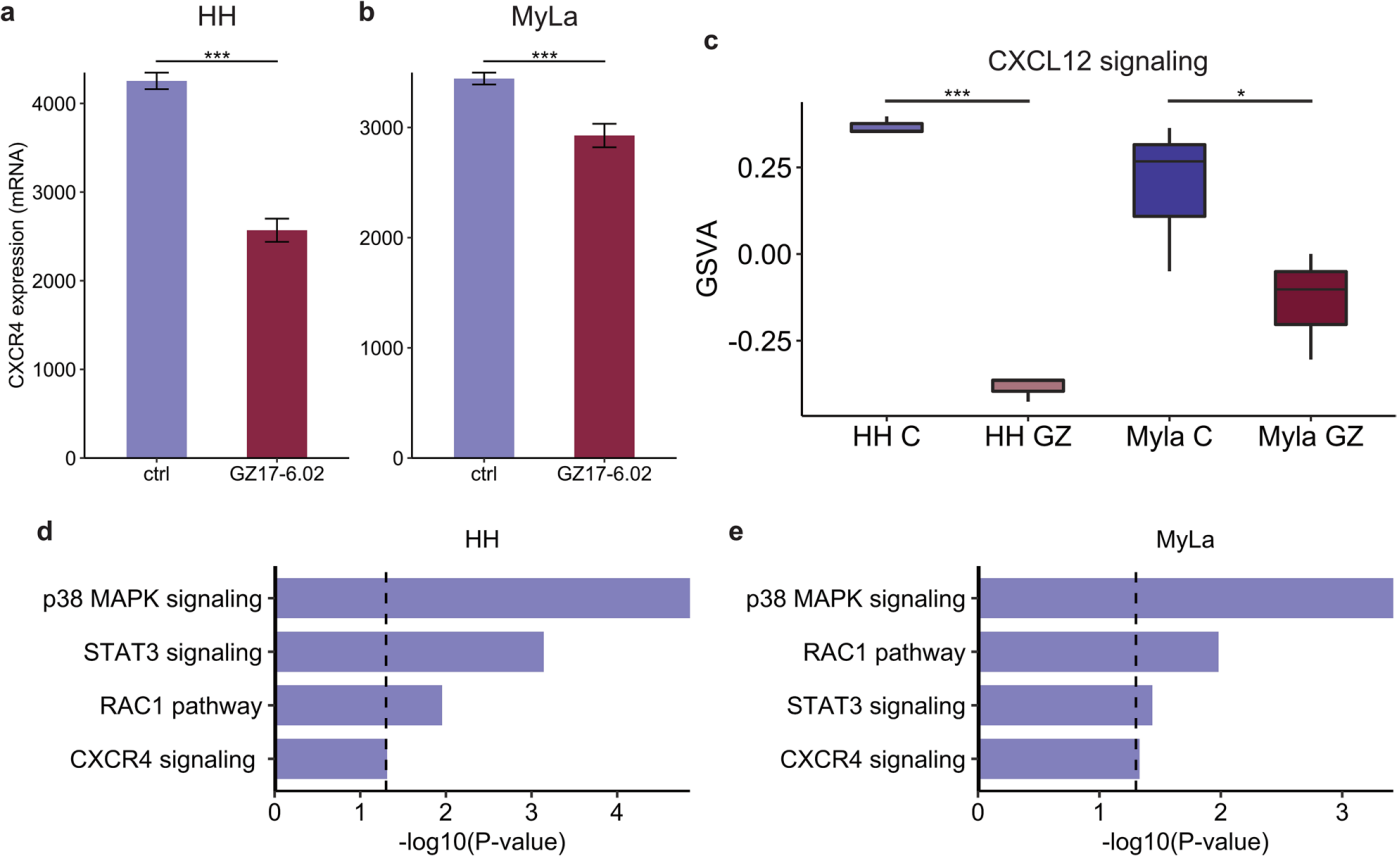
GZ17-6.02 suppresses the CXCR4/CXCL12 axis. (**A**) CXCR4 expression in control and GZ17-6.02 treated HH cells. (**B**) CXCR4 expression in control and GZ17-6.02 treated MyLa cells. (**C**) GSVA for CXCL12 signaling pathway in control and GZ17-6.02 treated HH and MyLa cells. (**D**) Hallmark EnrichR enrichment analysis of phospho-proteomic data showing suppression of CXCR4 signaling and downstream pathways in GZ17-6.02 treated HH cells. (**E**) Hallmark EnrichR enrichment analysis of phospho-proteomic data showing suppression of CXCR4 signaling and downstream pathways in GZ17-6.02 treated MyLa cells. *p < .05; ***p < .001; ctrl, control; GSVA, gene set variation analysis; GZ, GZ17-6.02.

It has previously been shown that suppression of Pi3K/Akt/mTOR signaling in CTCL decreases chemotaxis of malignant cells by disrupting the C-X-C chemokine receptor (CXCR)4/C-X-C motif chemokine ligand (CXCL)12 axis^52^. Given the suppression of Pi3K/Akt/mTOR signaling identified by our transcriptomic and proteomic analyses, we theorized that GZ17-6.02 would have a similar effect. Analysis of RNAseq data for changes in CXCR4 expression revealed a significant decrease in CXCR4 levels in both cell lines (*p* < 0.001 for HH and MyLa; Figs. 4A,B) after GZ17-6.02 treatment. Pathway analysis using GSVA also revealed a significant decreased in CXCL12 signaling in both cell lines (*p* < 0.001 for HH and *p* = 0.045 for MyLa; Figs. 4C). We next conducted an EnrichR enrichment analysis using gene lists of phosphoproteins downregulated by GZ17-6.02 to evaluate the compound’s effect on CXCR4/CXCL12 signaling and downstream pathways at a proteomic level (Figs. 4D,E)^57–59^. This analysis revealed suppression of p38 mitogen-activated protein kinases (MAPK), STAT3, RAC1, and CXCR4 signaling pathways in both cell lines.

### GZ17-6.02 inhibits the growth of MF in vivo

**Figure 5:**
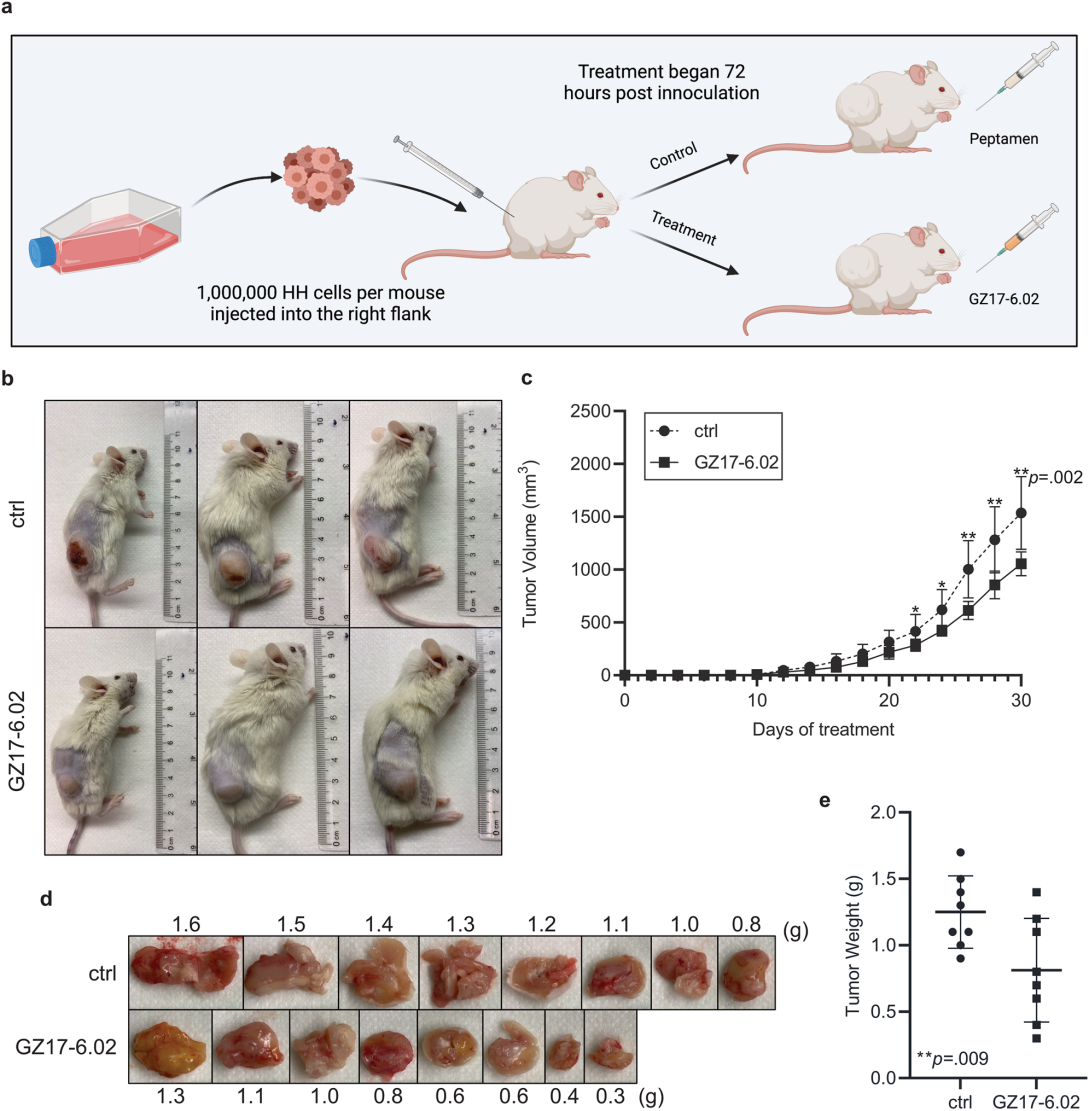
GZ17-6.02 inhibits MF growth in vivo. (**A**) Overview of experimental design. (**B**) Representative images of control and GZ17-6.02 treated mice after 30 days of treatment. (**C**) Tumor volume over time in control and GZ17-6.02 treated mice. (**D**) Representative images of tumors extracted from mice after 30 days of treatment. (**E**) Tumor weight for control and GZ17-6.02 treated mice. *p < .05; **p < .01; ctrl, control.

With the observed efficacy of GZ17-6.02 against MF cell lines in vitro*,* the next step was to asses the ability of the compound to inhibit MF growth in vivo using a subcutaneous tumor model. An overview of the experimental design is shown in Fig. 5A, while representative images of control and GZ17-6.02 treated mice after 30 days of treatment are shown in Fig. 5B. After 30 days, mice receiving GZ17-6.02 demonstrated a significant decrease in tumor volume (*p* = 0.002; Fig. 5C) and tumor weight (*p* = 0.009; Fig. 5D,E).

### Proteomics effects of GZ17-6.02 in vivo

**Figure 6:**
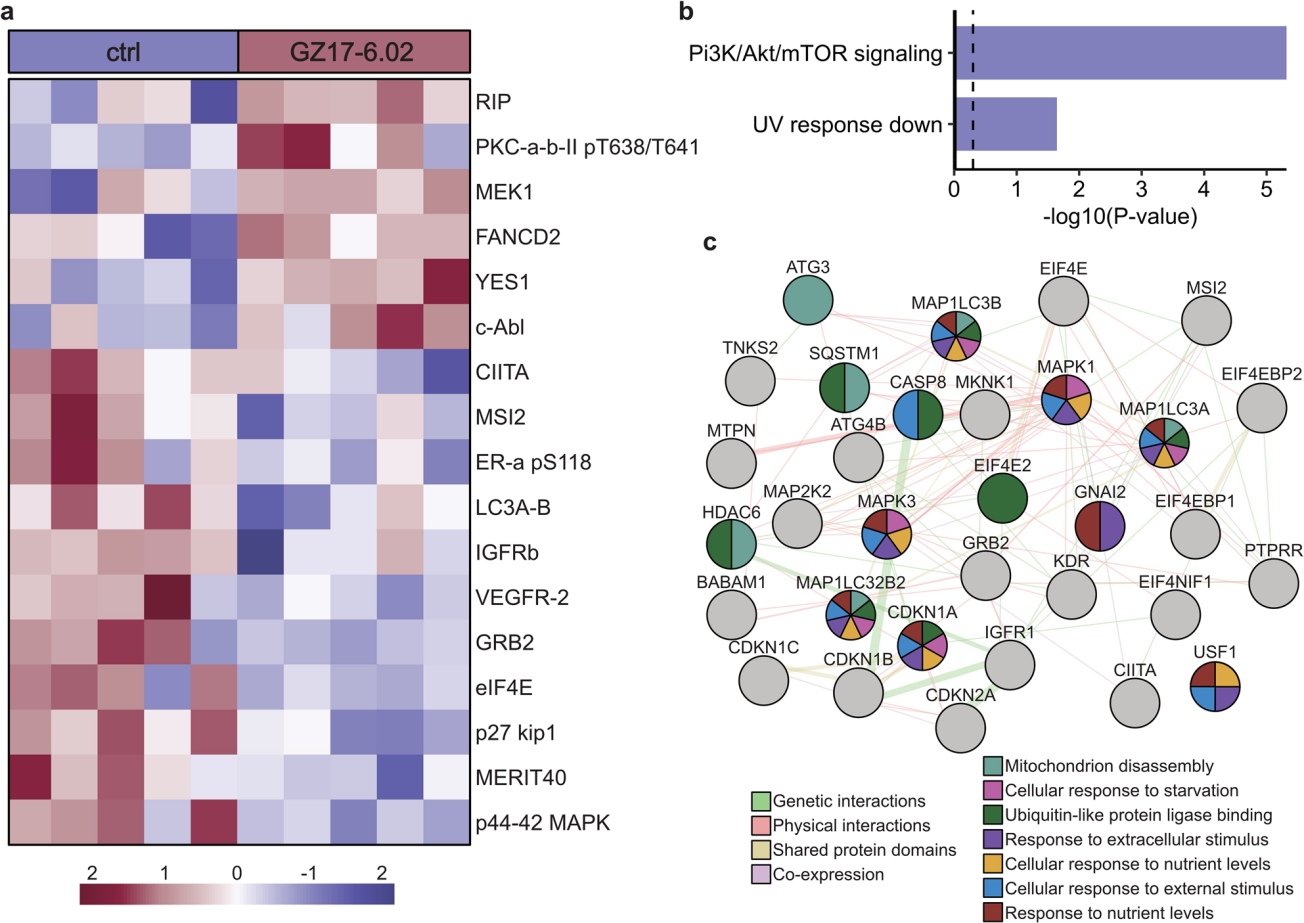
Proteomic effects of GZ17-6.02 in vivo. (**A**) Heatmap of significantly different proteins identified by RPPA between control and GZ17-6.02 treated mice. (**B**) Hallmark EnrichR enrichment analysis of proteins downregulated by GZ17-6.02 treatment. (**C**) Genemania network of gene lists of proteins downregulated by GZ17-6.02 showing proteins with genetic and physical integrations and that have shared protein domains or are co-expressed with the targets of GZ17-6.02. ctrl, control; UV, ultraviolet.

We next performed RPPA on tumors from control and GZ17-6.02 treated mice to gain a better understanding of the mechanism behind GZ17-6.02’s efficacy against MF, and determine is similar pathways that were identified as GZ17-6.02 targets in our in vitro analyses were affected in the subcutaneous tumor model. Figure 6A shows a heatmap of the significantly different proteins between control and GZ17-6.02 treated mice. Interestingly, GZ17-6.02 suppressed the expression of several proteins that may promote CTCL growth, including MAPK1, MAPK3, growth factor receptor bound protein (GRB)2, and mediator of RAP80 interactions and targeting subunit of 40 kDa (MERIT40). We next conducted an EnrichR functional enrichment analysis on gene lists of proteins downregulated by GZ17-6.02. Similar to our in vitro results, this analysis showed suppression of Pi3K/Akt/mTOR signaling in treated mice (Fig. 6B). We also constructed a GeneMANIA network using gene lists of proteins downregulated by GZ17-6.02 to highlight genes with shared protein domains and physical interactions or that are co-localized or co-expressed with the targets of GZ17-6.02 (Fig. 6C). We found that the most over-represented functions of these proteins were related to mitochondrion disassembly, response to nutrient levels and extracellular stimuli, and ubiquitin-like protein ligase binding.

## Discussion

Due to GZ17-6.02’s demonstrated efficacy against a variety of neoplastic conditions in vitro and in mouse models, the compound has recently gathered a great deal of attention as a possible therapeutic agent for a variety of neoplastic conditions^22–24,60–62^. As a combination agent, GZ17-6.02 may benefit from its ability to inhibit components of multiple signal pathways, and its components may be synergistic in their ability to slow tumor growth^20,63^. More recently, GZ17-6.02 has shown efficacy against cutaneous neoplasms such as actinic keratoses, cutaneous squamous cell carcinoma, and melanoma^21,26,64^. However, it has yet to be evaluated for the treatment of CTCL. Here, we assess GZ17-6.02 ability to inhibit MF growth in vitro and in a subcutaneous tumor model as well as further clarify its mechanism of action using transcriptomic and proteomic approaches. We demonstrate that the compound is a potent inhibitor of MF growth and induces apoptosis in MF cells. Mechanistically, GZ17-6.02 downregulates pathways crucial to MF growth and survival, such as NF-κB, Pi3k/Akt/mTOR, and MAPK pathways, while also suppressing the CXCR4/CXCL12 axis.

A previous studies evaluating GZ17-6.02’s anti-tumor properties have demonstrated its pro-apoptotic activity against head and neck squamous cell carcinoma cells which was mediated through cleavage of PARP and is constant with our findings^20^. In this study, we found that the compounds mechanism of action against MF is related to suppression of key targets in Pi3K/Akt/mTOR, mTORC1, and NF-κB signaling pathways. These findings are consistent with previous studies showing that curcumin suppresses the Pi3K/Akt/mTOR pathway and inhibits NF-κB signaling in various malignancies^65–70^. Furthermore, GZ17-6.02 itself is shown to suppress expression of mTORC1 and AKT proteins in vitro and in murine models and the topical formulation of the medication, GZ21T, was shown to inhibits the Pi3K/Akt pathway in a UVB-induced model of skin carcinogenesis^19,25,26,71^. These findings are notable and may partially explain the anti-tumor activity seen here as components of the Pi3k/Akt/mTOR pathway are among the most commonly mutated targets in human cancers^72^, and serve to promote the metabolism, proliferation, motility, and survival of malignant cells^73^. In CTCL specifically, immunohistochemistry (IHC) has demonstrated persistent activation of components of this pathway, such as p-Akt, p-70S6K, and p-eukaryotic initiation factor 4E-binding protein (4E-BP1)^74–76^. Furthermore, levels of these proteins showed a negative correlation with disease-free survival in advanced CTCL cases. Additionally, mTOR and dual Akt/mTOR inhibitors have shown efficacy in treating CTCL in murine models^52,77,78^. Similarly, the NF-κB pathway is also commonly dysregulated in CTCL^79^. Activating mutations and amplifications in upstream components, such as transforming growth factor beta-activated kinase (TAK)1, tumor necrosis factor receptor (TNFR)2, and SHANK associated RH domain-interacting protein (SHARPIN), have been identified in CTCLs and serve to promote the proliferation, migration, and invasion of malignant cells, while also exerting anti-apoptotic activities^80–82^. Furthermore, therapeutic agents targeted against the NF-κB pathway are shown to inhibit growth and induce apoptosis of CTCL cells^83,84^.

GZ17-6.02 was also found to suppress the CXCR4/CXCL12 axis in both HH and MyLa cells. Although GZ17-6.02’s effects on this CXCR4/CXCL12 signaling have not previously been evaluated, curcumin has been shown to suppress these pathways in various malignancies including endometrial and colon cancers^85,86^ Prior studies have shown that therapeutic agents targeting the Pi3K/Akt/mTOR pathway reduce CXCL12 mediated migration of CTCL and chronic lymphocytic leukemia cells^52,77^. As such, GZ17-6.02’s ability to suppress the CXCR4/CXCL12 axis may be due to its inhibition of Pi3K/Akt/mTOR as it was found to be a potent inhibitor of this pathway here and in prior studies^23,26^. These findings are noteworthy as CXCR4 expression is persistently increased in all stages of MF and serves to promote skin homing of malignant cells through interactions with CXCL12 produced by MF-associated cutaneous fibroblasts^87–89^. The CXCR4/CXCL12 axis also activates TORC1 to stimulate the proliferation of malignant T cells and promotes resistance to chemotherapeutic agents^52,88^. The findings here suggest that GZ17-6.02 may inhibit skin homing in MF patients and may show synergy with other chemotherapeutic agents as it has been shown to potentiate the effects of medications such as palbociclib, axitinib, doxorubicin, and pemetrexed^21,22,24,61,62^.

It is also worth noting that GZ17-6.02 was more effective at inducing apoptosis and suppressing the CXCR4/CXCL12 axis in HH than MyLa cells. The exact mechanism behind these differences is not currently understood but is likely due to the fact that the cell lines originate from different patients and exhibit molecular heterogeneity that may alter their response to therapeutics. These findings are in line with previous studies demonstrating that medications like vorinostat differentially modulate the expression of chemokine receptors and ligands in HH and MyLa cell lines^90^, and with the clinical observation that many therapeutics may only produce desired clinical outcomes in small percentages of MF patients^91,92^.

In vivo data demonstrate a significant reduction in tumor burden among mice treated with GZ17-6.02. The ability of the compound to reduce tumor growth in vivo as an oral formulation has previously been demonstrated in murine models of glioblastoma and pancreatic ductal adenocarcinoma^19,23^. Similar to our in vitro data, GZ17-6.02 suppressed the Pi3k/Akt/mTOR pathway in the subcutaneous tumor model. Additionally, proteomic analyses demonstrated suppression of targets such as MAPK1, MAPK3, GRB2, MERIT40. The compounds ability to inhibit targets in the MAPK pathways have been described in several in vitro studies, but its effect on GRB2 and MERIT40 have yet to be reported^20,21^. The findings here are noteworthy as the MAPK pathway is commonly mutated in human cancers and promotes aberrant growth and proliferation of malignant cells^93,94^. In MF, gain of function mutations have been detected in components of this pathway such as ERK-1, and B-Raf^95,96^. Furthermore, IHC studies have shown that the activated form of ERK1/2 may be present in the nucleus of malignant T-cells in up to 53% of CTCL lesions^74^. Similarly, GRB2 is an adapter protein that promotes MAPK signaling through interactions with Sos1 and has also been shown to commonly be amplified in CTCL cases^97,98^. Finally, MERIT40 is a downstream effector of the Pi3k/Akt/mTOR pathway that complexes with breast cancer gene, (BRCA)-1 in response to DNA damage to promote DNA repair, chemotherapeutic resistance, and the survival of malignant cells^99^. Taken together, our findings demonstrate that GZ17-6.02 affects a wide range of cellular targets, and its ability to inhibit MF growth is likely multifactorial.

Limitations of this study include its small sample size and testing only in mice. Additionally, experiments were not performed to determine the compound’s effect on pro- and anti-apoptotic molecules other than PARP, nor were experiments done to detect these targets in tissue from the in vivo experiments. Furthermore, the subcutaneous tumor model utilized here does not perfectly replicate the cutaneous biology of MF. As such, the compound’s full effect on skin homing of malignant cells cannot ascertained from this study. We theorize that it may decrease skin homing based on its ability to suppress the CXCR4/CXCL12 axis, but additional experiments utilizing varied in vivo MF models, including patient derived xenografts, should be performed. Additionally, experiments were not performed to assess the compounds effect on normal circulating T-cells, and further work should be done to determine if it may have adverse effects on healthy cells. In conclusion, GZ17-6.02 inhibits the growth of MF by modulating key drivers of multiple pathways such as MAPK, Pi3K/Akt/mTOR, and NF-κB signaling and represents a promising therapeutic option for the treatment of this disease. While few reliable animal models for patch and plaque stage MF exist, these preclinical studies help to pave the way for future clinical trials to study the efficacy of GZ17-6.02 and its topical analog, GZ21T, in the treatment of mycosis fungoides.

### Supplementary Information

Supplementary Information 1.

Supplementary Information 2.

## Data Availability

The data that support the findings of this study are available from the corresponding author, SGK, upon reasonable request.

## Abbreviations

MF: Mycosis fungoides
CTCL: Cutaneous T-cell lymphoma
CD: Cluster of differentiation
CCR: C-C chemokine receptor
EGFR: Epidermal growth factor receptor
Pi3K: Phosphoinositide 3-kinase
NF: Nuclear factor
STAT: Signal transducer and activator of transcription
ERK: Extracellular signal-regulated kinase
IACUC: Institutional Animal Care & Use Committee
DMSO: Dimethyl sulfoxide
PBS: Phosphate-buffered saline
PVDF: Polyvinylidene difluoride
TBS: Tris-buffered saline
TBS-T: Tris-buffered saline with 0.1% Tween-20
PARP: Poly (ADP-ribose) polymerase
HRP: Horseradish peroxidase
BSA: Bovine serum albumin
RNAseq: RNA sequencing
GSEA: Gene set enrichment analysis
GSVA: Gene set variation analysis
DEGs: Differentially expressed genes
RPPA: Reverse phase protein array
SDS: Sodium dodecyl sulfate
NSG: NOD SCID gamma
cPARP: Cleaved PARP
TNF: Tumor necrosis factor
mTORC: Mammalian target of rapamycin complex
mTOR: Mammalian target of rapamycin
CXCR: C-X-C chemokine receptor
CXCL: C-X-C motif chemokine ligand
MAPK: Mitogen-activated protein kinases
GRB: Growth factor receptor bound protein
MERIT40: Mediator of RAP80 interactions and targeting subunit of 40 kDa
IHC: Immunohistochemistry
4E-BP1: Eukaryotic initiation factor 4E-binding protein
TAK: Transforming growth factor beta-activated kinase
TNFR: Tumor necrosis factor receptor
SHARPIN: SHANK associated RH domain-interacting protein
BRCA: Breast cancer gene

## References

[1] Dummer R (2021). Cutaneous T cell lymphoma. Nat. Rev. Dis. Primers.

[2] Jawed SI, Myskowski PL, Horwitz S, Moskowitz A, Querfeld C (2014). Primary cutaneous T-cell lymphoma (mycosis fungoides and Sézary syndrome): part II. Prognosis, management, and future directions. J. Am. Acad. Dermatol..

[3] Agar NS (2010). Survival outcomes and prognostic factors in mycosis fungoides/sézary syndrome: Validation of the Revised International Society for Cutaneous Lymphomas/European Organisation for Research and Treatment of Cancer Staging Proposal. J. Clin. Oncol..

[4] Maguire A (2020). Early-stage mycosis fungoides: Epidemiology and prognosis. Acta Derm. Venereol..

[5] Howard MS, Smoller BR (2000). Mycosis fungoides: Classic disease and variant presentations. Semin Cutan Med. Surg..

[6] Larocca C, Kupper T (2019). Mycosis fungoides and sézary syndrome: An update. Hematol. Oncol. Clin. North. Am..

[7] Choi J (2021). Racial and ethnic disparities in inpatient health care utilization for mycosis fungoides: A cross-sectional analysis of the 2012–2017 National Inpatient Sample. J. Am. Acad. Dermatol..

[8] Demierre MF, Gan S, Jones J, Miller DR (2006). Significant impact of cutaneous T-cell lymphoma on patients' quality of life: Results of a 2005 National Cutaneous Lymphoma Foundation Survey. Cancer.

[9] Ottevanger R (2022). Itch in patients with cutaneous T-cell lymphoma as a quality of life indicator. JAAD Int..

[10] Kaul S (2019). Comorbidities in mycosis fungoides and racial differences in co-existent lymphomatoid papulosis: A cross-sectional study of 580 patients in an urban tertiary care center. Medicines.

[11] Huang AH (2019). Racial disparities in the clinical presentation and prognosis of patients with mycosis fungoides. J. Natl. Med. Assoc..

[12] Rowe B, Yosipovitch G (2016). Malignancy-associated pruritus. Eur. J. Pain.

[13] Trautinger F (2017). European Organisation for Research and Treatment of Cancer consensus recommendations for the treatment of mycosis fungoides/Sézary syndrome—Update 2017. Eur. J. Cancer.

[14] Sethi TK, Montanari F, Foss F, Reddy N (2021). How we treat advanced stage cutaneous T-cell lymphoma—mycosis fungoides and Sézary syndrome. Br. J. Haematol..

[15] Kaye FJ (1989). A randomized trial comparing combination electron-beam radiation and chemotherapy with topical therapy in the initial treatment of mycosis fungoides. N. Engl. J. Med..

[16] Willemze R, Hodak E, Zinzani PL, Specht L, Ladetto M (2018). Primary cutaneous lymphomas: ESMO Clinical Practice Guidelines for diagnosis, treatment and follow-up. Ann. Oncol..

[17] Kamijo H, Miyagaki T (2021). Mycosis fungoides and sézary syndrome: Updates and review of current therapy. Curr. Treat Options Oncol..

[18] Cerroni L (2018). Mycosis fungoides-clinical and histopathologic features, differential diagnosis, and treatment. Semin Cutan Med. Surg..

[19] Ghosh C (2019). Super-enhancers: Novel target for pancreatic ductal adenocarcinoma. Oncotarget.

[20] Vishwakarma V (2018). Potent antitumor effects of a combination of three nutraceutical compounds. Sci. Rep..

[21] Booth L, West C, Von Hoff D, Kirkwood JM, Dent P (2021). GZ17–6.02 Interacts With [MEK1/2 and B-RAF Inhibitors] to Kill Melanoma Cells. Front. Oncol..

[22] Booth L, West C, Moore RP, Von Hoff D, Dent P (2022). GZ17-6.02 and palbociclib interact to kill ER + breast cancer cells. Oncotarget.

[23] Choi J (2022). GZ17-6.02 inhibits the growth of EGFRvIII + Glioblastoma. Int. J. Mol. Sci..

[24] Booth L, West C, Moore RP, Hoff DV, Dent P (2022). GZ17-6.02 and axitinib interact to kill renal carcinoma cells. Oncotarget.

[25] Bordeaux ZA, Kwatra SG, Booth L, Dent P (2023). A novel combination of isovanillin, curcumin, and harmine (GZ17-6.02) enhances cell death and alters signaling in actinic keratoses cells when compared to individual components and two-component combinations. Anticancer Drugs.

[26] Bordeaux ZA (2023). Topical GZ21T inhibits the growth of actinic keratoses in a UVB-induced model of skin carcinogenesis. JID Innov..

[27] Lin SS (2009). Curcumin inhibits the migration and invasion of human A549 lung cancer cells through the inhibition of matrix metalloproteinase-2 and -9 and Vascular Endothelial Growth Factor (VEGF). Cancer Lett..

[28] Wu SH (2010). Curcumin induces apoptosis in human non-small cell lung cancer NCI-H460 cells through ER stress and caspase cascade- and mitochondria-dependent pathways. Anticancer Res..

[29] Unlu A, Nayir E, Dogukan Kalenderoglu M, Kirca O, Ozdogan M (2016). Curcumin (Turmeric) and cancer. J. Buon.

[30] Maiti P, Scott J, Sengupta D, Al-Gharaibeh A, Dunbar GL (2019). Curcumin and solid lipid curcumin particles induce autophagy, but inhibit mitophagy and the PI3K-Akt/mTOR pathway in cultured glioblastoma cells. Int. J. Mol. Sci..

[31] Chen A, Xu J, Johnson AC (2006). Curcumin inhibits human colon cancer cell growth by suppressing gene expression of epidermal growth factor receptor through reducing the activity of the transcription factor Egr-1. Oncogene.

[32] Zhen L (2014). Curcumin inhibits oral squamous cell carcinoma proliferation and invasion via EGFR signaling pathways. Int. J. Clin. Exp. Pathol..

[33] Trochopoulos AGX (2020). Antineoplastic effect of a novel nanosized curcumin on cutaneous T cell lymphoma. Oncol. Lett..

[34] Zhang C (2010). Curcumin selectively induces apoptosis in cutaneous T-cell lymphoma cell lines and patients' PBMCs: Potential role for STAT-3 and NF-kappaB signaling. J. Invest. Dermatol..

[35] Pozo N (2013). Inhibition of DYRK1A destabilizes EGFR and reduces EGFR-dependent glioblastoma growth. J. Clin. Investig..

[36] Chamcheu JC (2019). Role and therapeutic targeting of the PI3K/Akt/mTOR signaling pathway in skin cancer: A review of current status and future trends on natural and synthetic agents therapy. Cells.

[37] Gao J (2017). Harmine suppresses the proliferation and migration of human ovarian cancer cells through inhibiting ERK/CREB pathway. Oncol. Rep..

[38] Zhang L, Li D, Yu S (2020). Pharmacological effects of harmine and its derivatives: A review. Arch Pharm. Res..

[39] Nafie E (2021). Harmine inhibits breast cancer cell migration and invasion by inducing the degradation of Twist1. PLoS ONE.

[40] He J (2022). Harmine suppresses breast cancer cell migration and invasion by regulating TAZ-mediated epithelial-mesenchymal transition. Am. J. Cancer Res..

[41] Kersey PJ (2012). Ensembl genomes: An integrative resource for genome-scale data from non-vertebrate species. Nucleic Acids Res..

[42] Dobin A (2013). STAR: Ultrafast universal RNA-seq aligner. Bioinformatics.

[43] Love MI, Huber W, Anders S (2014). Moderated estimation of fold change and dispersion for RNA-seq data with DESeq2. Genome Biol..

[44] Huber W (2015). Orchestrating high-throughput genomic analysis with Bioconductor. Nat. Methods.

[45] Mootha VK (2003). PGC-1α-responsive genes involved in oxidative phosphorylation are coordinately downregulated in human diabetes. Nat. Genetics.

[46] Subramanian A (2005). Gene set enrichment analysis: A knowledge-based approach for interpreting genome-wide expression profiles. Proc. Natl. Acad. Sci. U S A.

[47] Liberzon A (2015). The Molecular Signatures Database (MSigDB) hallmark gene set collection. Cell Syst..

[48] Hänzelmann S, Castelo R, Guinney J (2013). GSVA: Gene set variation analysis for microarray and RNA-Seq data. BMC Bioinform..

[49] Kuleshov MV (2016). EnrichR: A comprehensive gene set enrichment analysis web server 2016 update. Nucleic Acids Res..

[50] Chen EY (2013). EnrichR: Interactive and collaborative HTML5 gene list enrichment analysis tool. BMC Bioinform..

[51] Xie Z (2021). Gene set knowledge discovery with EnrichR. Curr. Protoc..

[52] Bresin A (2020). Preclinical evidence for targeting PI3K/mTOR signaling with dual-inhibitors as a therapeutic strategy against cutaneous T-cell lymphoma. J. Invest. Dermatol..

[53] Krejsgaard T (2010). A novel xenograft model of cutaneous T-cell lymphoma. Exp. Dermatol..

[54] Huang Y, Su MW, Jiang X, Zhou Y (2015). Evidence of an oncogenic role of aberrant TOX activation in cutaneous T-cell lymphoma. Blood.

[55] Tewari M (1995). Yama/CPP32 beta, a mammalian homolog of CED-3, is a CrmA-inhibitable protease that cleaves the death substrate poly(ADP-ribose) polymerase. Cell.

[56] Kaufmann SH, Desnoyers S, Ottaviano Y, Davidson NE, Poirier GG (1993). Specific proteolytic cleavage of poly(ADP-ribose) polymerase: An early marker of chemotherapy-induced apoptosis. Cancer Res..

[57] Gao D, Sun H, Zhu J, Tang Y, Li S (2018). CXCL12 induces migration of Schwann cells via p38 MAPK and autocrine of CXCL12 by the CXCR4 receptor. Int. J. Clin. Exp. Pathol..

[58] Liu X (2014). Activation of STAT3 is involved in malignancy mediated by CXCL12–CXCR4 signaling in human breast cancer. Oncol. Rep..

[59] Zoughlami Y (2012). Regulation of CXCR4 conformation by the small GTPase Rac1: Implications for HIV infection. Blood.

[60] Booth L, Roberts JL, West C, Dent P (2022). GZ17-6.02 kills prostate cancer cells in vitro and in vivo. Front. Oncol..

[61] Booth L, West C, Moore RP, Von Hoff D, Dent P (2021). GZ17–6.02 and pemetrexed interact to kill osimertinib-resistant NSCLC cells that express mutant ERBB1 proteins. Front. Oncol..

[62] Booth L, West C, Von Hoff D, Dent P (2021). Corrigendum: GZ17-6.02 and doxorubicin interact to kill sarcoma cells via autophagy and death receptor signaling. Front. Oncol..

[63] Jänne PA, Gray N, Settleman J (2009). Factors underlying sensitivity of cancers to small-molecule kinase inhibitors. Nat. Rev. Drug Discov..

[64] Bordeaux ZA, Kwatra SG, Booth L, Dent P (2022). A novel combination of isovanillin, curcumin, and harmine (GZ17-6.02) enhances cell death and alters signaling in actinic keratoses cells when compared to individual components and two-component combinations. Anti-cancer Drugs.

[65] Qiao Q, Jiang Y, Li G (2013). Inhibition of the PI3K/AKT-NF-κB pathway with curcumin enhanced radiation-induced apoptosis in human Burkitt’s lymphoma. J. Pharmacol. Sci..

[66] Borges GA (2020). Curcumin downregulates the PI3K-AKT-mTOR pathway and inhibits growth and progression in head and neck cancer cells. Phytother. Res..

[67] Kuttikrishnan S (2019). Curcumin induces apoptotic cell death via inhibition of PI3-kinase/AKT pathway in B-precursor acute lymphoblastic leukemia. Front. Oncol..

[68] Aggarwal BB, Gupta SC, Sung B (2013). Curcumin: An orally bioavailable blocker of TNF and other pro-inflammatory biomarkers. Br. J. Pharmacol..

[69] Vadhan-Raj S (2007). Curcumin downregulates NF-kB and related genes in patients with multiple myeloma: Results of a phase I/II study. Blood.

[70] Xu YX, Pindolia KR, Janakiraman N, Chapman RA, Gautam SC (1997). Curcumin inhibits IL1 alpha and TNF-alpha induction of AP-1 and NF-kB DNA-binding activity in bone marrow stromal cells. Hematopathol. Mol. Hematol..

[71] Booth L, West C, Hoff DV, Dent P (2020). GZ17-6.02 and doxorubicin interact to kill sarcoma cells via autophagy and death receptor signaling. Front. Oncol..

[72] Weigelt B, Downward J (2012). Genomic determinants of PI3K pathway inhibitor response in cancer. Front. Oncol..

[73] Manning BD, Toker A (2017). AKT/PKB signaling: Navigating the network. Cell.

[74] Levidou G (2013). A comprehensive immunohistochemical approach of AKT/mTOR pathway and p-STAT3 in mycosis fungoides. J. Am. Acad. Dermatol..

[75] Marzec M (2008). IL-2- and IL-15-induced activation of the rapamycin-sensitive mTORC1 pathway in malignant CD4 + T lymphocytes. Blood.

[76] Cristofoletti C (2019). Blood and skin-derived Sezary cells: Differences in proliferation-index, activation of PI3K/AKT/mTORC1 pathway and its prognostic relevance. Leukemia.

[77] Blunt MD (2015). The PI3K/mTOR inhibitor PF-04691502 induces apoptosis and inhibits microenvironmental signaling in CLL and the Eµ-TCL1 mouse model. Blood.

[78] Kremer M, Sliva K, Klemke CD, Schnierle BS (2010). Cutaneous T-cell lymphoma cells are sensitive to rapamycin. Exp. Dermatol..

[79] Izban KF (2000). Constitutive expression of NF-kappa B is a characteristic feature of mycosis fungoides: Implications for apoptosis resistance and pathogenesis. Hum. Pathol..

[80] Ungewickell A (2015). Genomic analysis of mycosis fungoides and Sézary syndrome identifies recurrent alterations in TNFR2. Nat. Genet..

[81] Chen B, Zheng Y, Zhu J, Liang Y (2019). SHARPIN overexpression promotes TAK1 expression and activates JNKs and NF-κB pathway in mycosis fungoides. Exp. Dermatol..

[82] Gallardo F (2018). Novel phosphorylated TAK1 species with functional impact on NF-κB and β-catenin signaling in human Cutaneous T-cell lymphoma. Leukemia.

[83] Juvekar A (2011). Bortezomib induces nuclear translocation of IκBα resulting in gene-specific suppression of NF-κB—Dependent transcription and induction of apoptosis in CTCL. Mol. Cancer Res..

[84] Sors A (2006). Down-regulating constitutive activation of the NF-kappaB canonical pathway overcomes the resistance of cutaneous T-cell lymphoma to apoptosis. Blood.

[85] Zhang Z (2016). Curcumin inhibits tumor epithelial-mesenchymal transition by downregulating the Wnt signaling pathway and upregulating NKD2 expression in colon cancer cells. Oncol. Rep..

[86] Sirohi VK (2017). Curcumin exhibits anti-tumor effect and attenuates cellular migration via Slit-2 mediated down-regulation of SDF-1 and CXCR4 in endometrial adenocarcinoma cells. J. Nutr. Biochem..

[87] Maj J (2015). Expression of CXCR4 and CXCL12 and their correlations to the cell proliferation and angiogenesis in mycosis fungoides. Postepy Dermatol. Alergol..

[88] Aronovich A (2021). Cancer-associated fibroblasts in mycosis fungoides promote tumor cell migration and drug resistance through CXCL12/CXCR4. J. Invest. Dermatol..

[89] Narducci MG (2006). Skin homing of Sézary cells involves SDF-1-CXCR4 signaling and down-regulation of CD26/dipeptidylpeptidase IV. Blood.

[90] Bordeaux ZA (2023). Differential response of mycosis fungoides cells to vorinostat. Int. J. Mol. Sci..

[91] Olsen EA (2007). Phase IIb multicenter trial of vorinostat in patients with persistent, progressive, or treatment refractory cutaneous T-cell lymphoma. J. Clin. Oncol..

[92] Lowe MN, Plosker GL (2000). Bexarotene. Am. J. Clin. Dermatol..

[93] Dhillon AS, Hagan S, Rath O, Kolch W (2007). MAP kinase signalling pathways in cancer. Oncogene.

[94] Braicu C (2019). A comprehensive review on MAPK: a promising therapeutic target in cancer. Cancers.

[95] da Silva Almeida AC (2015). The mutational landscape of cutaneous T cell lymphoma and Sézary syndrome. Nat. Genet..

[96] Choi J (2015). Genomic landscape of cutaneous T cell lymphoma. Nat. Genet..

[97] Lin WM (2012). Characterization of the DNA copy-number genome in the blood of cutaneous T-cell lymphoma patients. J. Invest. Dermatol..

[98] Qu Y (2014). SUMOylation of Grb2 enhances the ERK activity by increasing its binding with Sos1. Mol. Cancer.

[99] Brown KK, Montaser-Kouhsari L, Beck AH, Toker A (2015). MERIT40 Is an Akt substrate that promotes resolution of DNA damage induced by chemotherapy. Cell Rep..

