# Supplementary figures and images for: Transcriptomic and proteomic analysis of tumor suppressive effects of GZ17-6.02 against mycosis fungoides
Source: Sci Rep. 2024 Jan 23;14:1955. doi: 10.1038/s41598-024-52544-z (PMC10805783; doi:10.1038/s41598-024-52544-z)

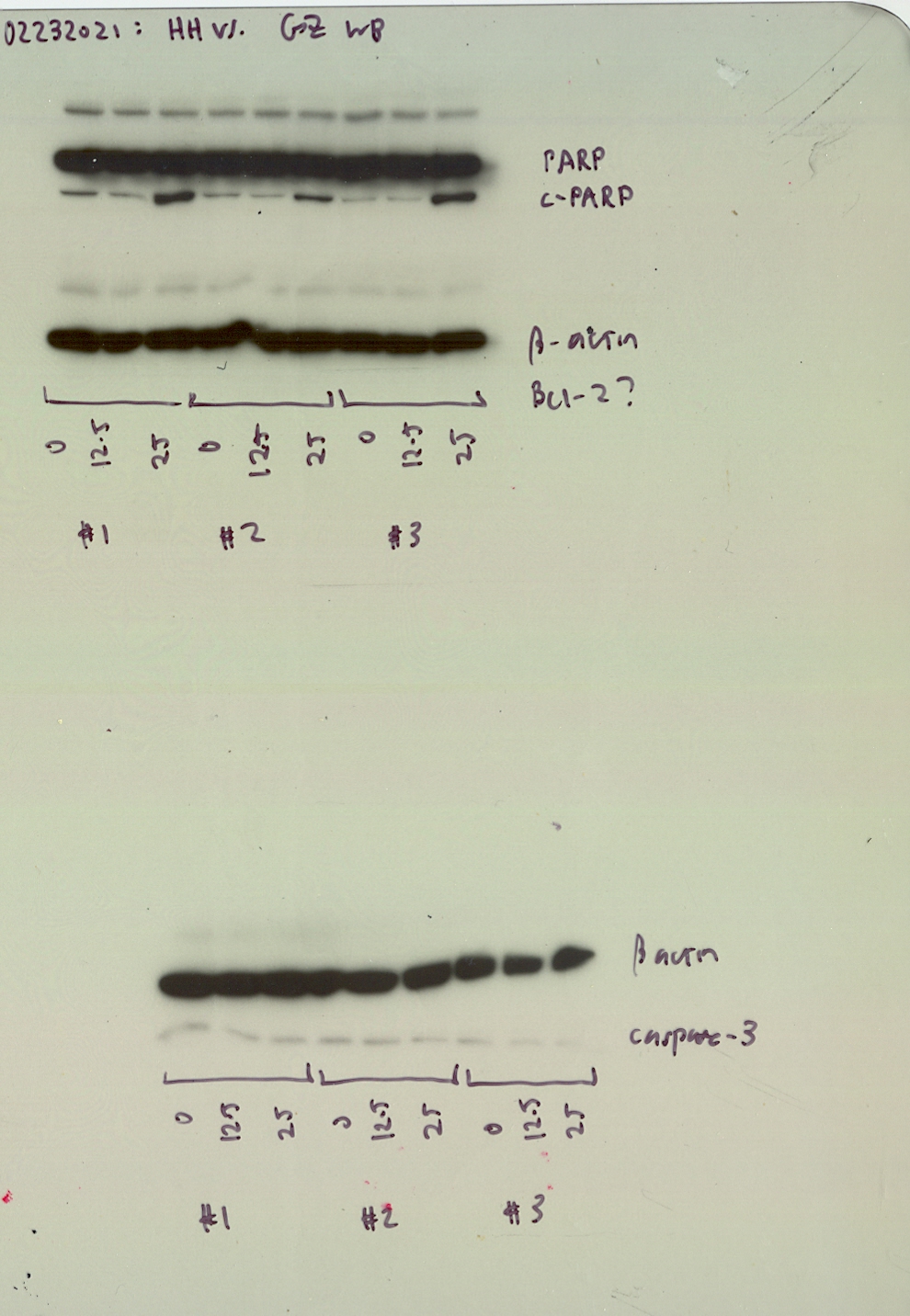

Supplement: Supplementary file 1 — Supplementary Information 1. [file 41598_2024_52544_MOESM1_ESM.jpg]

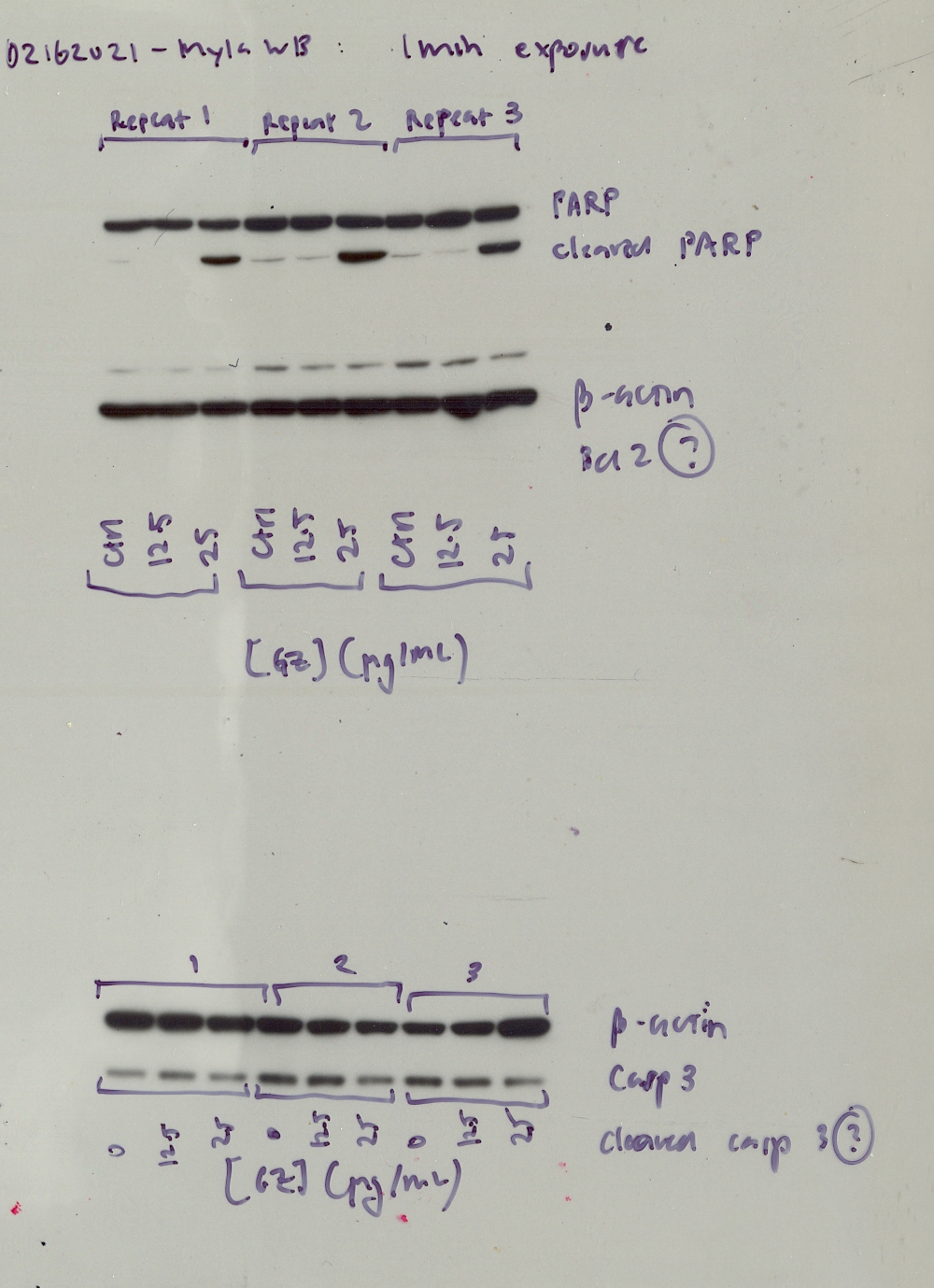

Supplement: Supplementary file 2 — Supplementary Information 2. [file 41598_2024_52544_MOESM2_ESM.jpg]
